# It Is Better for Younger Workers: The Gain Cycle between Job Crafting and Work Engagement

**DOI:** 10.3390/ijerph192114378

**Published:** 2022-11-03

**Authors:** Gabriela Topa, Mercedes Aranda-Carmena

**Affiliations:** 1 Faculty of Psychology, Universidad Nacional de Educación a Distancia, 28040 Madrid, Spain; 2 Universidad Autónoma de Chile, Santiago de Chile 7500000, Chile; 3 Faculty of Health Sciences, Department of Psychology, Universidad Rey Juan Carlos, 28922 Madrid, Spain

**Keywords:** aging, crafting behavior, employees’ age, engagement

## Abstract

Job Crafting has been proposed as a new perspective, consisting in a bottom-up strategy to achieve person–job fit by emphasizing employees’ active participation and spontaneous change in job design, which is specifically adequate for older workers. Despite this fact, the cyclical influence between Work Engagement and Job Crafting over time has been less researched. We postulated that a gain cycle could be observed in the relationships between Job Crafting and its outcomes. Hence, we tested a longitudinal moderated mediation model in which Work Engagement increases over time through an increment in Job Crafting behaviors (Hypothesis 1), while this process is moderated by workers’ age (Hypothesis 2). The present study follows a three-wave design where participants (*N* = 126) responded to online surveys at three measurement waves, three months apart. At Time 1 and Time 3, we assessed Work Engagement, Job Crafting behavior, and demographic variables, while at Time 2 we only assessed Job Crafting. Our findings partially differ from what was expected. The findings supported that the relationship between Work Engagement at Time 1 and changes in Job Crafting behavior across time was negative and non-significant, failing to provide support for Hypothesis 1. Related to Hypothesis 2, our results are mixed. Although the interaction between changes in Job Crafting and workers’ age did not demonstrate a statistical influence on Work Engagement at Time 3, our findings suggested that the direct influence was complemented by a negative indirect effect through the longitudinal increase of Job Crafting, which mainly affects aged workers. Practical and theoretical implications are discussed.

## 1. Introduction

The aging of the working population implies a relevant challenge for societies, organizations, and human resource managers [1] that could also be considered an opportunity for further development. Specifically for organizations, there are at least three main topics to be considered. The first one would be managing the diversity of the workforce. Since age diversity highly increased during the last decades, differences in available resources and specific demands from younger and older workers should be taken into account in order to maintain sustainable work ability and organizational performance [2]. The second one should be maintaining motivation at work. As the literature strongly supports that younger and older workers seem to be motivated by different types of goals and interests [3,4], organizational leaders should consider these differences when proposing tasks and offer rewards both to younger and older employees, in order to maintain their Work Engagement over time. Finally, empirical evidence highly demonstrates that some age-related changes would affect individual performance, such as changes in intelligence, emotions, and health [5,6]. Hence, organizational strategies for adapting job characteristics to older workers are recommendable [7]. However, beyond organizational design, individual workers themselves could proactively modify their jobs in order to achieve a better person–environment fit [8], and one of the most relevant strategies for doing that is Job Crafting. Previous research support that Job Crafting would be considered a useful way to adapt job features to individual skills and necessities that improve Work Engagement [9]. Moreover, it is necessary to understand not only if workers would craft their jobs, but *when* or *under what conditions* this strategy works. Based on these considerations, we aim to achieve three goals with this study. First, we report on the cyclical influence between Work Engagement and Job Crafting over time. Second, we empirically investigate how changes in Job Crafting over time could affect later Work Engagement. Finally, we deeply explore potential differences between younger and older workers in the influence of Job Crafting over time on work-related variables. Via an age-heterogenous sample, we also generalize the results to the whole working age range.

### 1.1. Job Crafting and Work Engagement Relationships

Job Crafting has been proposed as a new perspective [9], consisting in a bottom-up strategy to achieve Person–job fit by emphasizing employees’ active participation and spontaneous change in job design, which is considered a desirable demonstration of proactivity. While Wrzesniewski and Dutton [10] initially described Job Crafting as consisting of task changes, cognitive changes, and relational changes, the most widely known theoretical proposal includes it as a component of the Job Demands Resources Model (JD-R) [11]. Under this approach, the dimensions of Job Crafting have been defined as follows: (1) increasing structural job resources (i.e., crafting the job autonomy or job variety); (2) increasing social job resources (i.e., crafting advice or support from colleagues); (3) increasing challenging job demands (i.e., crafting for increasing demands that imply difficult goals and professional development), and (4) decreasing hindering job demands (i.e., crafting fewer job demands if the worker feels overwhelmed). As Tims et al. [12] hypothesized, Job Crafting could be a consequence of Work Engagement. Work Engagement can be defined as positive work-related behavior or a positive state of mind at work characterized by vigor, dedication, and absorption. Vigor refers to high levels of energy and mental resilience while working, the willingness to invest effort in one’s work, and persistence even in the face of difficulties. Dedication refers to being strongly involved in one’s work, and experiencing a sense of significance, enthusiasm, inspiration, pride, and challenge; and absorption refers to being fully concentrated and happily engrossed in one’s work, whereby time passes quickly and one has difficulties with detaching oneself from work [13]. Hence, as employees with high levels of Work Engagement are energetic, dedicated to, and immersed in their work, they would be intrinsically motivated to meet their work goals, and more probably would activate or search for resources to achieve them. At the same time, Tims et al. [14] also suggested that Job Crafting would influence both future resources and demands, and it would also later impact Work Engagement. Empirical evidence supported the two hypotheses showing that Work Engagement could predict Job Crafting [15] and Job Crafting could be an antecedent of Work Engagement [16,17,18].

### 1.2. Job Crafting Gain Cycle

Job Crafting has been investigated separately as an antecedent and consequence of Work Engagement, but the theoretical approach based on the JD-R model also suggests that there are reciprocal relationships in the model. The interaction between Work Engagement and Job Crafting suggests that they are mutually reinforcing. Some studies have provided empirical evidence for the mediating role of Job Crafting in the relationships between Work Engagement and other work-related variables [19,20]. While other studies propose the mediating role of Job Crafting in the relationships between Work Engagement and job-related outcomes [21]. Despite this growing body of evidence, as recommended by one of the latest meta-analyses [9], more research is needed on the cyclical effects of Job Crafting on certain variables, such as Work Engagement itself.

Theoretically, as postulated by the conservation of resources (COR) theory [22], engaged employees are committed to avoiding losses and increasing the accumulation of valuable resources, especially to be able to perform their job better, and Job Crafting would be one way to do this. By pushing the boundaries of their work, engaged workers can actively seek additional resources to enable them to perform their tasks better and avoid burnout. In addition, Job Crafters can constantly improve their resource pools, preventing them from depleting in the future, and increase positive job outcomes by maintaining job engagement, which in turn could sustain subsequent Job Crafting. In other words, we expect employees who are generally highly committed to their work to show higher levels of Job Crafting. In the same vein, those who do crafting might be strongly committed to their work. Thus, having COR as an umbrella, we expect there to be a gain cycle in which job engagement and job crafting reinforce each other over time.

### 1.3. Job Crafting and Ageing

Different proposals have been done about the relationships between Job Crafting and aging. On the one hand, some authors [23] suggested that younger workers would be more oriented to development goals, thus applying Job Crafting as a way to obtain knowledge and expertise at work, e.g., increasing their hindrance demands [24]. On the other hand, some authors suggested that aged workers would be more oriented to Job Crafting due to their necessity to adapt to the age-related decrease in skills and health. In this vein, aged workers could reduce their demands or even try to increase their social resources, for example asking for help from their younger counterparts [25].

More recent empirical research fueled the debate on the differences in Job Crafting behaviors as a function of workers’ age. For instance, De Lange et al. [26] found a negative and significant relationship between age and some kinds of Job Crafting behaviors, while others remain positive but non-significant. In line with this finding, Nagy et al. [27] revealed a negative and weak relation between age and overall Job Crafting. Considering the influence of other variables, such as subjective age, some studies showed negative relationships with Job Crafting over and above the effect of chronological age [28]. Later, Bashir and colleagues [29] proposed that Job Crafting could have different roles as a function of employees’ age. They found that younger workers adopt Job Crafting while old-age employees did not engage in Job Crafting behaviors, perhaps because aged workers give more relevance to intrinsic job characteristics, which could be difficult to alter by crafting.

One of the most comprehensive meta-analyses on Job Crafting [9] also revised the empirical evidence on the differences in Job Crafting as a function of employees’ age. The authors proposed that older workers, having accumulated knowledge and expertise, could be more oriented to apply Job Crafting as a strategy for adapting their jobs than younger workers. At the same time, they also acknowledge that experience would be associated with the development of cognitive routines that do not promote changes at work. Hence, it would be possible that younger workers apply Job Crafting more frequently than their older counterparts [30]. Their results finally showed a negative relationship between age and Job Crafting, showing that more research is needed to better understand the role of Job Crafting as a function of workers’ age. Hence, based on this mixed evidence, we proposed that employees’ age could moderate the relationships between early Work Engagement and later engagement mediated by the increase in Job Crafting.

Based on the above-reviewed literature, in the present study, the following hypotheses are proposed:

**Hypothesis** **1** **(H1).** *Job Crafting will mediate the relationship between Work Engagement over time.*

**Hypothesis** **2** **(H2).** *Job Crafting will mediate the relationship between Work Engagement over time, and conditionally to the levels of age.*

**Figure 1:**
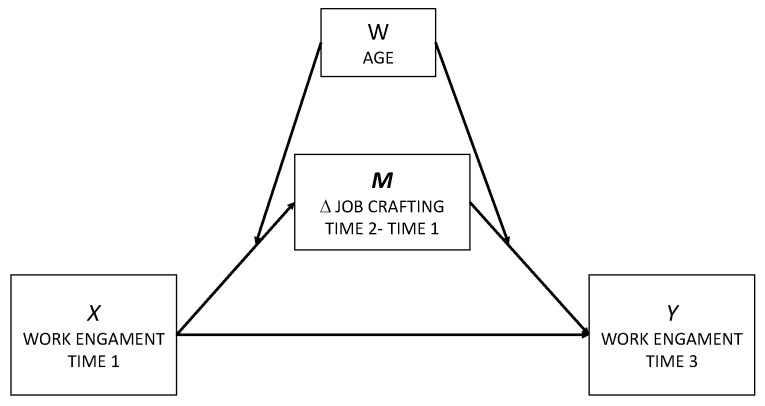
Global model for this study applying the Model nº 58 from Hayes.

The global model proposed by our study is depicted in Figure 1.

## 2. Materials and Methods

### 2.1. Design and Participants

The present study follows a three-wave design where participants (*N* = 126) responded to online surveys at three measurement waves, three months apart. We employed a time lag of three months between Time 1 and Time 2, and another three months between Time 2 and Time 3 based on the recent recommendation of taking “the shortest time-lag needed to capture a particular effect, with the least possible intrusion to the natural process” [31]. Our participants were employed in a wide range of jobs (e.g., nurse, teacher, administrative officer, physicians, taxi drivers, etc.) and areas of the economy, such as education, hospital, banking, marketing, and telecommunication, in Spain. Most of our participants were women, (*N* = 82) 65% of the whole sample. Participants’ age ranged from 20 years to 63, with 8% age ranged from 20 years to 30.40% age ranged from 40 years to 50, and 35.2% age ranged from 50 years to 63 years. Their mean working experience was 14.17 years (S.D. = 8.17), while the mean organizational tenure was 10.6 years (S.D. = 8.46) and most of them worked in the same organization for 6 years and more (58.7%). We used an age-diverse sample, following the procedure suggested by previous studies as that of Zacher and Rudolph [32], since researchers have suggested that chronological age should be treated as a moderator in analyses instead of limiting artificially the range of age including only younger or older workers [33].

### 2.2. Instruments

At Time 1 and 3, we assessed Work Engagement, and demographic variables, while Job Crafting was assessed at Time 1 and Time 2.

Work Engagement (Time 1 and Time 3): it has been assessed using the UWES-3 (Utrecht Work Engagement Scale) [34]. This ultra-short version offers the advantage of reducing the length of two-wave surveys. The original study reported reliability of Cronbach’s Alpha = 0.77 for the Spanish form, but recent studies [35] reported higher values as α = 0.94. Each item covered one of the dimensions of Work Engagement and an overall engagement score can be calculated. The items are: *At my work, I feel bursting with energy* (vigor); *I am enthusiastic about my job* (dedication); and *I am immersed in my work* (absorption). In the present study, reliability was α = 0.77 at Time 1 and α = 0.87 at Time 3. The items were scored on a five-point Likert scale ranging from 1 (*Never*) to 5 (*Always*).

Job Crafting (Time 1 and Time 2): it was assessed with the Spanish short version of the Job Crafting Scale, originally developed by Tims and colleagues [12]. While the original scale contains 21 items, the reduced version only includes 12 in the Spanish language. The four dimensions of the original scale have been represented by three items each: Increasing structural job resources (i.e., *I try to develop my capabilities*), Decreasing hindering demands (i.e., *I try to ensure that my work is mentally less intense*), Increasing social job resources (i.e., *I ask my supervisor to coach me*), and Increasing challenging demands (i.e., *When an interesting project comes along, I offer myself proactively as project co-worker*). This short version maintains adequate reliability values ranging from α = 0.64 to α = 0.78 in the original study [36]. In the present study, reliability was α = 0.70 at Time 1 and α = 0.78 at Time 2. The items were scored on a five-point Likert scale ranging from 1 (*Never*) to 5 (*Always*).

Demographic characteristics were assessed at Time 1: age (as chronological age or years), gender, educational level, and organizational tenure, as well as the type of job and area of the economy where the respondents work. Even though there is wide debate on the operationalization of a “younger” versus “older” worker in empirical research, older or aged workers are defined commonly as 45-plus [37,38]. In the same vein, the United States’ Bureau of Labor Statistics usually considers >45 years as a cut point for aged workers. Based on this, in the present study, we created a dichotomic age variable grouping our participants as younger employees (less than 45 years) and older employees (45 plus).

Confirmatory factor analyses were conducted. Structural equation modeling with maximum-likelihood estimation was used with the raw data as the entry. Several measures of model fit have been recommended, such as a small chi-squared (χ^2^) and non-significant p-value. Other tests of model fit were the root mean square error of approximation (RMSEA), and comparative fit index (CFI). Models are considered to fit the data well when the following criteria are met: *p* > 0.05, χ^2^/df < 5, RMSEA < 0.06, TLI > 0.95 and CFI > 0.95. A first CFA was applied to Work Engagement Time 1 and Time 3. The test statistics for the initial model (χ^2^ = 66.44, df = 8, *p* < 0.001; χ^2^/df = 8.31) were unsatisfactory. Modification indices and standardized residuals indicated that correlation between absorption item at Time 1 and Time 3 should be added, resulting in an improved model. Adding the correlation between the two vigor items led to the final model with a reasonable fit to the data (χ^2^ = 9.815, df = 6, *p* < 0.133, χ^2^/df = 1.636, RMSEA = 0.071 and CFI = 0.993). A second CFA was applied to Job Crafting Time 1 and Time 2. The test statistics for the initial model (χ^2^ = 1121.94, df = 251, *p* < 0.001; χ^2^/df = 4.47) were unsatisfactory. Modification indices and standardized residuals indicated that correlation between the same items at Time 1 and Time 2 should be added, resulting in the final model with a reasonable fit to the data (χ^2^ = 589.515, df = 239, *p* < 0.001, χ^2^/df = 2.467)**.**

### 2.3. Procedure

Participants were recruited with the collaboration of students enrolled in an Occupational Risk Prevention Master, who received in exchange academic credits, following the procedure suggested by Demerouti and Rispens, [39] about student-recruited samples. Convenience sampling was used to recruit participants that fulfilled the following inclusion criteria. The inclusion criteria for the participants were as follows: (a) above 18 years old; (b) resident in Spain; (c) currently working full time or part-time; (d) able to read and understand the survey, and (e) have Internet access. Data were collected during 2020 and 2021 after the COVID-19 sanitary crisis in Spain. Each student recruited ten participants and provided them with an anonymous code in order to fulfill the Time 1 survey. Three months later, students contacted the participants again, asking them to fill in the questionnaire once more, and they contacted them again three months later. For Times 1, 2, and 3 participants were paired using anonymous codes. At the beginning of each survey, potential participants were informed about the research objectives, anonymity, voluntariness, and the possibility of leaving the study at any time, and the research group do not know any personal data from the participants. All the participants had initially signed informed consent and then completed the survey by a Google forms questionnaire. Concerning ethical standards for research, the study adhered to the latest version of the World Medical Association’s Declaration of Helsinki revised in Fortaleza [40]. The National Distance Education University Bio-Ethical Committee approved this research.

### 2.4. Statistical Analyses

To determine bivariate correlations, Pearson’s correlation coefficient analyses were conducted.

The hypotheses were tested using the Process macro for SPSS designed by Hayes [41]. We performed the model nº 58 testing a moderated mediational model. In this model, both paths, from the independent variable (Work Engagement Time 1, X) to the mediator (increase of Job Crafting between Time 1 and Time 2, M) and from the mediator (M) to the dependent variable (Work Engagement Time 3, Y), were moderated by the same variable (employees’ age, W). Employees’ age was dichotomized as younger (aged below 45 years) and older workers (aged above 45 years). The results about conditional indirect effects were obtained by calculating 5000 bootstrap samples for bias-corrected bootstrap confidence intervals (CIs) with a 95% level of confidence intervals.

As the use of difference scores in empirical research has been extensively criticized [42], as they could be affected by potential unreliability, systematic correlation with their components, and spurious correlation with other variables, we applied the procedure named ENREF_30, suggested by Smith and Beaton [43]. In order to calculate the mediator, Job Crafting increase longitudinally, we regressed Time 2 scores of Job Crafting on the Time 1 Job Crafting scores, obtaining the Timer 2 minus Time 1 changes in Job Crafting as the standardized residual scores. The positive residual scores revealed an increase in Job Crafting behaviors and the negative scores showed a decrease. These values have been entered into the regression analyses as a mediator variable between Work Engagement in Time 1 and Work Engagement in Time 3.

We conducted a preliminary check about differences among variables between male and female participants, as well as respondents categorized as a function of age. We found no statistically significant differences in the means and variances of the variables as a function of gender. Despite this fact, we found two differences: (i) male participants showed less Work Engagement both at Time 1 and Time 3 (Time 1 Males M = 3.85 vs. Time 1 Females M = 3.97; and Time 3 Males M = 3.74 vs. Time 3 Females M = 3.94); and (ii) while males showed an increase in Job Crafting behavior between Time 1 and Time 2, females showed a decrease (Male M = 0.040 vs. Female M = −0.023). Testing for differences in the means and variances as a function of age, we found only one statistically significant difference between participants aged below 45 years and those aged above 45 years (F = 5.19, *p* = 0.024). In particular, younger workers showed an increase in Job Crafting behavior between Time 1 and Time 2 (M = 0.14), while older workers showed a decrease (M = −0.29).

## 3. Results

### 3.1. Descriptive and Correlational Analyses

Descriptive analyses showed that there is an overall decrease in Work Engagement between Time 1 and Time 3, while the scores deviation increases (Table 1). Related to Job Crafting, the scores revealed stability between Time 1 and Time 2, with a slight increase in deviation of the scores. Considering Pearson’s correlation matrix, age is negative and significantly related to Job Crafting at Time 2, while negative but not significantly related to Work Engagement at Time 1 and positively but not significantly related to Work Engagement at Time 3.

### 3.2. Testing Hypotheses

In testing our hypotheses, we computed the model for the mediator variable (M) Job Crafting (∆ = standardized residual scores). Initially, we checked for gender, which was not significant (Table 2). Unexpectedly, Work Engagement at Time 1 negatively predicted Job Crafting behavior changes between Time 1 and Time 2 (*b* = −0.3812, *p* = 0.3102). But as previous support to our hypothesis 2, age and the interaction term (computed as a product of age and Work Engagement Time 1) have respectively a negative (*b* = −2.9554, *p* < 0.05) and positive (*b* = 0.6845, *p*< 0.05) significant effect on change in Job Crafting between T1 and T2, and both effects were statistically significant. This finding suggests that Work Engagement at Time 1 negatively impacts Job Crafting changes but particularly for older workers, while its negative influence would be less for younger.

The second model has been calculated for Work Engagement at Time 3, showing that gender has no significant effect and Work Engagement at Time 1 strongly predicted Work Engagement at Time 3 (*b* = 0.7846, *p* < 0.001). Contrary to our hypothesis 1, and despite its positive effect, ∆Job Crafting (M) failed to significantly predict Work Engagement at Time 3 (*b* = 0.2053, *p* = 0.1348) as well as the interaction term computed between Age and ∆Job Crafting (M) (*b* = −0.1638, *p* = 0.0769), which only reached a tendency value.

**Figure 2:**
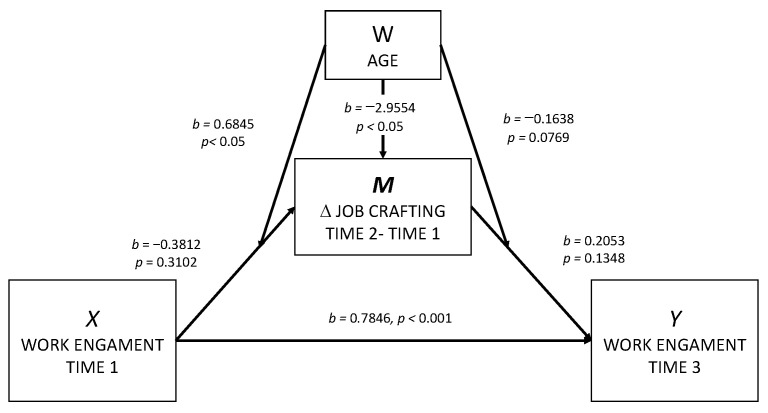
Main findings for this study applying the Model nº 58 from Hayes.

Considering the direct effect of Work Engagement Time 1 (*X*) on Work Engagement Time 3 (*Y*), it was positive and statistically significant (*b* = 0.7846, SE = 0.0676, *t* = 11.61, *p* < 0.001, 95% CI [0.6508, 0.9183]). When we computed the conditional indirect effects by bootstrapped analyses, we found partial support for Hypothesis 2. While the influence of Work Engagement at Time 1 on Work Engagement at Time 3, mediated by the changes in Job Crafting between Time 1 and Time 2, was positive and non-significant for younger workers (*b* = 0.0111, 95% CI [−0.0100, 0.0607]), a negative impact was found for older workers (*b* = −0.1119, 95% CI [−0.3391, 0.0048]). Moreover, the index of moderated mediation is intended to serve as an effect size/coefficient for whether the moderator affects the overall indirect effect. The 95% confidence interval for the index of moderated mediation did not contain zero (*b* = −0.1230, SE = 0.0853, 95% CI: [−0.3529, −0.0028]), suggesting that there were differences between the indirect effects at the two levels of the moderator. At one level, the effect was positive, but at the other level, it was negative. Our findings mean that the direct influence of Work Engagement at Time 1 on Work Engagement at Time 3 was complemented by a negative indirect effect through the longitudinal increase of Job Crafting, which mainly affects aged workers. The findings are displayed in Figure 2.

## 4. Discussion

### 4.1. Main Findings

The present study had three main goals. First, we reported our findings on the cyclical influence between Work Engagement and Job Crafting over time. Second, we empirically investigated how changes in Job Crafting over time could affect later Work Engagement. Finally, we deeply explored potential differences between younger and older workers in the influence of Job Crafting over time on work-related variables.

Our findings partially differ from what was expected [44], allowing us to discuss the complex processes of a self-perpetuating influence between Job Crafting and Work Engagement over time. Regarding the influence of Work Engagement at Time 1 on Job crafting changes over time, our results have been unexpected. The findings supported that the relationship between Work Engagement Time 1 and changes in Job Crafting behavior across time was negative and non-significant, failing to provide support for Hypothesis 1. Moreover, the impact of changes in Job Crafting behavior on Work Engagement at Time 3 confirmed its positive influence despite its absence of statistical significance. Hence, our first hypothesis has not been supported. This finding partially coincides with the results from Goel and colleagues [45], where the relationship between Work Engagement at Time 1 and Collaborative Job Crafting at Time 2 was not significant.

A relevant point that can be discussed would be that the Job Crafting theoretical model adopted in the present study does not distinguish between approach crafting and avoidance crafting. Approach crafting entails those behaviors oriented to gain desirable outcomes, while avoidance crafting refers to actions focused on preventing undesirable outcomes, the three first subdimensions of the Job Crafting Scale represent approach crafting and the last includes avoidance crafting [46]. Subsequent research showed the nonlinear effects of these two forms of Job Crafting on outcomes. Some authors found that approach crafting positively impacts Work Engagement but not for free because it increases workload, which in turn promotes burnout over time. At the same time, avoidance crafting decreases job demands, reducing Work Engagement over time. In the same vein, the Boehnlein and Baum meta-analysis [47] showed that approach crafting promotes Work Engagement, while this association has not been found for avoidance crafting. Since in the present study the two forms of Job Crafting (approach and avoidance) have been combined in a global index, this differential effect of the two dimensions on Work Engagement could explain the present findings.

Considering that the JD-R model constantly showed empirical evidence of the positive influence of Work Engagement on Job Crafting [12], as well as the positive influence of Job Crating on Work Engagement [48], our results seem a bit disappointing. Job crafting behaviors have been proposed as a way to adapt job features to individuals’ needs and motives. This way would be specifically available for highly engaged employees, based on the proposal that those rich in resources would be more capable of orchestrating gain cycles. Perhaps less attention has been devoted to that investing in increasing their Job Crafting behaviors would not be the unique way for engaged workers. On the one hand, despite some level of proactivity, engaged workers could be high consciousness and perfectionists, and they could not be involved in expanding their Job Crafting behavior because they see them as distractors, which should be avoided. On the other, engaged workers could invest their resources in crafting their jobs, but only in order to reach some desired level of job performance. Hence, when this level is acquired, they could not deviate their attention by crafting, in order to maintain the adequate level of job performance recently reached. Future research should explore both ideas: Firstly, the potential moderating role of personality traits, such as perfectionism, in the relationships between Work Engagement and Job Crafting over time. Second, the potential moderating role of job performance in the relationships between Work Engagement and later implications in Job Crafting behavior.

In relation to Hypothesis 2, our results are mixed. Although the interaction between changes in Job Crafting and workers’ age did not reach statistical influence on Work Engagement at Time 3, the conditional indirect effects suggested that some relevant information should be taken into account. The point estimates for younger workers showed a positive influence, low and not statistically significant, but a negative influence and strong for older workers. Our results highlighted that employees’ age matters. As age increases, the negative effect of changes in Job Crafting over time on Work Engagement at Time 3 also increases. As motivation theories stated [49], and empirical research supported [50], older workers would be less attracted by developmental goals and a higher level of effort. Hence, the motivational pattern proposed by the JD-R Model would be more prominent for younger than for older workers. Contrary to previous findings [51], our results did not show a gain cycle for aged workers. Instead, we found a loss cycle statistically significant for older workers.

Future research should deeply explore other moderators that would have interactive effects with age, such as career development opportunities. Despite being older, some types of jobs entail the opportunity for further career advancements, while others are not, being less motivating for employees since they perceive a career plateau. The interaction between career advancement opportunities and age could differentially affect aged employees and their motivation to engage in Job Crafting behaviors. Our sample is highly heterogenous, and it could affect our findings due to the differences in possibilities of Job Crafting behaviors as a function of the type of job and occupation. Some aged employees could not engage in Job Crafting due to their lack of motivation based on their perceptions that their job does not permit any adaptation, for instance, a clerical job in Public Administration.

### 4.2. Limitations and Practical Implications of the Present Study

The present study has some limitations. Firstly, although our methodology for analyzing longitudinal data tried to avoid some criticism of the difference scores, we acknowledge that research on behavioral changes entails a lot of difficulties. Secondly, different time points during the year would have different signification as a function of the type of job. For instance, for teachers, the beginning of the school term could be more stressful, while tax day might discomfort accountants, or the summer for firefighters, affecting our assessment of changes in Job Crafting over time. Thirdly, our design only included three waves, and this would not be enough information to assess some kinds of behavioral changes adequately. We should consider that Job Crafting behavior could positively impact later Work Engagement [52]. Still, perhaps it could produce depletion of resources as a consequence of an investment in Job Crafting and the final result would be a further decrease in Work Engagement. Fourthly, even though a three-wave study provides us with more solid evidence on the relationships between the variables under study, more information should be necessary in order to firmly establish the type of rhythm of cyclical influence between Work Engagement and Job Crafting over time [53]. Related to this topic, there is also the time lag of three months between times, which was considered enough to capture the desired effect, but more theoretically grounded intervals would be recommended. Moreover, our sample is not representative and the possibility of generalization of our findings is very limited. In the same vein, evidence from a heterogenous sample regarding the type of jobs and occupations could preclude us from firmly concluding about the availability of Job Crafting opportunities.

In relation to the practical implications of the present study, they could be organized at the individual level, the group level, and the organizational level. Job Crafting is not the unique type of adaptative behavior aimed to address the misfit between the employee and his/her job. Considering our results, which suggest that the gain-cycle of Work Engagement over time through the increase in Job Crafting only was significant for younger workers, older employees could try to gain resources by crafting the boundaries of nonwork and work-related activities. Such a type of behavior, named time–spatial crafting, consists of an active selection of workplaces, work locations, or working windows of time that better fit job tasks and private demands. Hence, aged workers could emphasize this kind of crafting behavior at the individual level. At the group level, the crossover process has been proposed as a dyadic interindividual transmission of psychological states between the team members. As Hobfoll and colleagues stated [54], the crossover of Engagement could be observed when the vigor and dedication expressed by one member positively influenced another member. Given that this kind of crossover of resources is relevant for gain spirals, the crossover of Work Engagement would offer an opportunity to reduce misfit for aged workers through the mobilization of energetic resources. At the organizational level, age-diverse groups or departments offer the mechanism by which resource gains can be transferred from younger to older workers. Hence, organizations that adequately manage age diversity can facilitate frequent exchanges between colleagues that promote older workers who would be benefited from the gain cycle more common among younger workers [7].

## 5. Conclusions

This preliminary evidence from Job Crafting over time and its effects on Work Engagement among workers in Spain would contribute to the debate on the relationships between work-related attitudes and employees’ age. Despite those certain limitations, we think that our work highlighted that younger and older workers should not be treated in the same way, and that differential HRM practices would be adequate for different groups. Following such recent proposals on mature workers in organizations such as Parker and Andrei’s [7], broader approaches are needed in order to attract and retain mature workers, individualizing strategies with aim to fulfill their needs and motives, as well as integrate actions relevant for organizational outcomes.

## Figures and Tables

**Table 1:** Mean, standard deviation, and Pearson’s’ correlations between variables.

Variables	Mean	S.D.	1.	2.	3.	4.
1. Age (number of years)	42.21	8.703	1			
2. Work Engagement Time 1	3.93	0.69	−0.015	1		
3. Job Crafting Time 1	3.46	0.47	−0.194 *	0.495 **	1	
4. Job Crafting Time 2	3.47	0.51	−0.141	0.408 **	0.711 **	1
5. Work Engagement Time 3	3.87	0.73	0.126	0.728 **	0.459 **	0.486 **

*N* = 126, * *p* < 0.05; ** *p* < 0.01. S.D.: Standard Deviation.

**Table 2:** Direct and indirect effects of the model.

Variables	∆Job Crafting (M) *R*^2^ = 0.1594	Work Engagement Time 3 (Y) *R*^2^ = 0.5587
	*b* (SE)	*b* (SE)
Constant	2.1853 (1.5032)	0.4350 (0.3170)
Gender (1 male, 2 female)	−0.0875 (0.1768)	0.0948 (0.0935)
Work Engagement Time 1 (WE T1)	−0.3812 (0.3740)	0.7846 (0.0676) ***
Age	−2.9554 (1.1338) **	0.1387 (0.0973)
Interaction 1 (WE T1 × Age)	0.6485 (0.2858) *	
∆Job Crafting (M)		0.2053 (0.1348)
Interaction 2 (Age × M)		−0.1638 (0.0918) +
Conditional indirect effects of Work Engagement T1 through ∆Job Crafting (M)		**Work Engagement Time 3 (Y)**
		Point estimate (95% CI)
Younger workers (minus 45 years)		0.0111 (−0.0100, 0.0607)
Older workers (+45 years)		−0.1119 (−0.3391, 0.0048)
Index of moderated mediation	−0.1230 (0.0853)	−0.1230 [−0.3529, −0.0028]

*N* = 126. +—tendency value, * *p* < 0.05; ** *p* < 0.01; *** *p* < 0.001.

## Data Availability

The data presented in this study are available on request from the corresponding author. The data are not publicly available due to measures to protect participant confidentiality.
